# Parasites and Their Pedigree

**Published:** 1884-10

**Authors:** 


### PARASITES AND THEIR PEDIGREE.

COMMON tapeworm consists of a very minute head, attaching itself by suckers and hooks to a man's intestines; of a slender neck, and of hundreds of joints. Each joint is really a semi-independent animal; and the tapeworm is therefore a compound animal, and presents us with a colony of similar beings. A large tapeworm may measure twenty or thirty feet; and new joints are continually being budded out from the head and neck. Hence the physician can never be sure that he has cured a case of tapeworm until he has seen the head and neck of the animal. If a man swallowed the egg of a tapeworm, he would not be infested thereby. The young worm has to pass its early life in the body of another warm-blooded animal; and in the case of a common tapeworm, it is "the gintleman that pays the rint" which acts the part of nurse or first host. Man, in other words, obtains the common tapeworm guest from the pig.

When this animal swallows the egg of a tapeworm, the young worm bursts through the egg-case and bores its way to the pig's muscles. If the porker is affected by numerous embroyos, that is, if it has swallowed a large number of eggs, it will become feverish and ill, and it will then be said to have developed measels. The "measels" of the pig are the visitation of young tapeworms. In the muscles of the pig, then, these young worms rest. *J'y suis; j'y reste,* is decidedly the motto of the young worm. It develops a little head and neck, and it also, by way of a tail, produces a little bladder or bag. If the pig dies a natural death, and is respectably interred, or if the pig should live long enough, these youthful tapeworms will respectively perish or will degenerate and disappear from the tissues of the aged porker.

But assuming that the usual Nemesis of the pig race overtakes the animal, then, in the form of pork, it will gladden the hearts of certain members of the human race. Now let us suppose that a man eats a portion of the "measly" pork; let us further suppose that the pork has been imperfectly cooked; then comes the "tide of fortune" to the young worms. For when the young worm has been eaten by the man, the bladder-tail drops off. Each little head and neck, finding itself in the human stomach, recognizes its lawful "habitat." Each attaches itself to the lining membrane of the human intestines, and each, by a process of budding, produces joint after joint, until man is presented with his matured "guest." The great lesson to be learnt from our survey of parasites, is care in the choice and increased care in the cooking of our food. It should be remembered that the germs of these parasites are killed by a sufficiently long exposure to heat. Hence, while underdone meat may have its charms it has likewise its grave dangers. Pork, in any and every fashion, should at all times be thoroughly cooked. In this latter case the parasitic horde may not merely be destroyed, but may even contribute, in a microscopic way, to human nutrition.

### ANGLO-SWISS MILK-FOOD.

DECIDED superiority is claimed for the Anglo-Swiss Milk-Food in comparison with any other farinaceous food for infants. No so-called milk-food consists entirely of milk; all are partly composed of cereal products, involving, when not properly prepared, the presence of an injurious amount of starch, which the highest authorities agree in condemning for young children. The Anglo-Swiss Condensed Milk Company overcomes this objectionable feature of milk food, as usually supplied, by meeting an essential requirement in the method of preparing it, so that when gradually heated with water, according to the directions for use, the starch contained in the materials used is converted, in a satisfactory degree, into soluble and easily digestible dextrine and into sugar.

The Anglo-Swiss Milk-Food has beed found to meet these essential conditions to the satisfaction of physicians and others who have taken the pains to examine it, and we invite critical examination of it in comparison with any other food.

